# The Role of Montelukast in the Prevention and Treatment of Capsular Contracture (Baker Grade III–IV) - A Systematic Review and Case Analysis

**DOI:** 10.1007/s00266-025-05583-4

**Published:** 2026-01-08

**Authors:** Camilla Prosenz, Judith Aschauer, Alice Thürlimann, Carlo M. Oranges, Daniel Kalbermatten, Paolo Montemurro, Mathias Tremp

**Affiliations:** 1 Faculty of Medicine, University of Basel, Basel, BS Switzerland; 2 Department of Gynecology, Zurich Hospital Zollikerberg, 8125 Zurich, Switzerland; 3 Department of Plastic Surgery, Solothurner Spitäler, Schöngrünstrasse 42, 4500 Solothurn, Switzerland; 4 Department of Plastic, Reconstructive and Aesthetic Surgery, Geneva University Hospitals (HUG), University of Geneva, Geneva, Switzerland; 5 Akademikliniken Stockholm, Storängsvägen 10, 11541 Stockholm, Sweden; 6 TREMP Plastic Surgery AG, Dorfplatz 1, 6330 Cham, Switzerland

**Keywords:** Implant capsular contracture, Drug interactions, Patient outcome assessment, Breast augmentation, Secondary prevention

## Abstract

**Introduction:**

Capsular contracture remains a significant challenge in breast implant surgery. Recent research has focused on the role of inflammatory mediators, particularly leukotrienes. The aim of this article was to conduct a systematic review of existing clinical data on an orally active selective leukotriene receptor antagonist (montelukast) and capsular contracture management in combination with surgery and to present some representative cases.

**Materials and Methods:**

A meta-analysis was performed with the following keywords: "Montelukast,” "Singulair," "capsular contracture," "Baker grade," and "breast implants." References from identified articles were screened to capture additional studies not retrieved through database searches (PubMed and Semantic Scholar). Moreover, we provided representative case samples in patients with Baker Grade III-IV capsular contracture undergoing surgical treatment with adjuvant montelukast treatment once daily for 90 days.

**Results:**

Five studies with 1343 patients were included. Prophylactic montelukast reduced capsular contracture rates, particularly in Baker grade I-II cases, with statistical significance in two studies. In Baker grade III-IV contracture, montelukast had limited effect, and most patients required surgery. Extended therapy downgraded some severe cases to Baker grade II. In line with the current literature, our case examples confirmed the efficacy of montelukast with minimal side effects.

**Conclusion:**

By synthesizing available evidence, the potential of leukotriene receptor antagonism as a therapeutic adjunct to surgery for the treatment of capsular contracture is promising. The findings could pave the way for new treatment paradigms in breast implant surgery, potentially improving outcomes and patient satisfaction.

**Level of Evidence V:**

This journal requires that authors assign a level of evidence to each article. For a full description of these Evidence-Based Medicine ratings, please refer to the Table of Contents or the online  Instructions to Authors  www.springer.com/00266.

## Introduction

Capsular contracture remains a significant challenge in breast implant surgery, characterized by the abnormal tightening of scar tissue around the implant. This condition, classified using the Baker scale from Grade I to IV, can result in breast firmness, discomfort, and aesthetic distortion, often necessitating revision surgery [1, 2].

The etiology of capsular contracture is complex and multifaceted, primarily driven by an inflammatory reaction. Factors contributing to this inflammatory response include infection, hematoma formation, and patient-specific epigenetic hyperreactions [3–5]. The type of implant surface, textured or smooth, also seems to play a role in the development of capsular contracture when placed subglandularly or subfascial according to some studies [6, 7]. That being said, no significant difference between implants was observed when the implants were placed submuscularly [6].

Recent research has focused on the role of inflammatory mediators, particularly leukotrienes, in the pathophysiology of capsular contracture. These molecules promote fibroblast activity and collagen deposition, contributing to the formation of the problematic fibrous capsule. This understanding has led to an interest in the potential use of leukotriene receptor antagonists, such as montelukast (Singulair®), in both the prevention and treatment of capsular contracture [8, 9]. Other agents, notably zafirlukast (Accolate®), have been explored; however, its use has declined after reports of hepatotoxicity and liver enzyme elevation, and montelukast is generally preferred for its safety profile [7, 8]. The potential use of montelukast in managing capsular contracture represents an exciting area of research that could significantly impact clinical practice. By offering a noninvasive option for prevention or treatment, it could address a critical need in breast implant surgery. However, standardized protocols and more comprehensive clinical studies are necessary to fully evaluate its efficacy and safety in this context. To date, there is a paucity of data in the literature on this topic. Thus, the aim of this article was to conduct a systematic review of existing clinical data on montelukast's role in capsular contracture management and present some representative cases with histological and radiological assessment.

## Methods

### Study Design

**Fig. 1:**
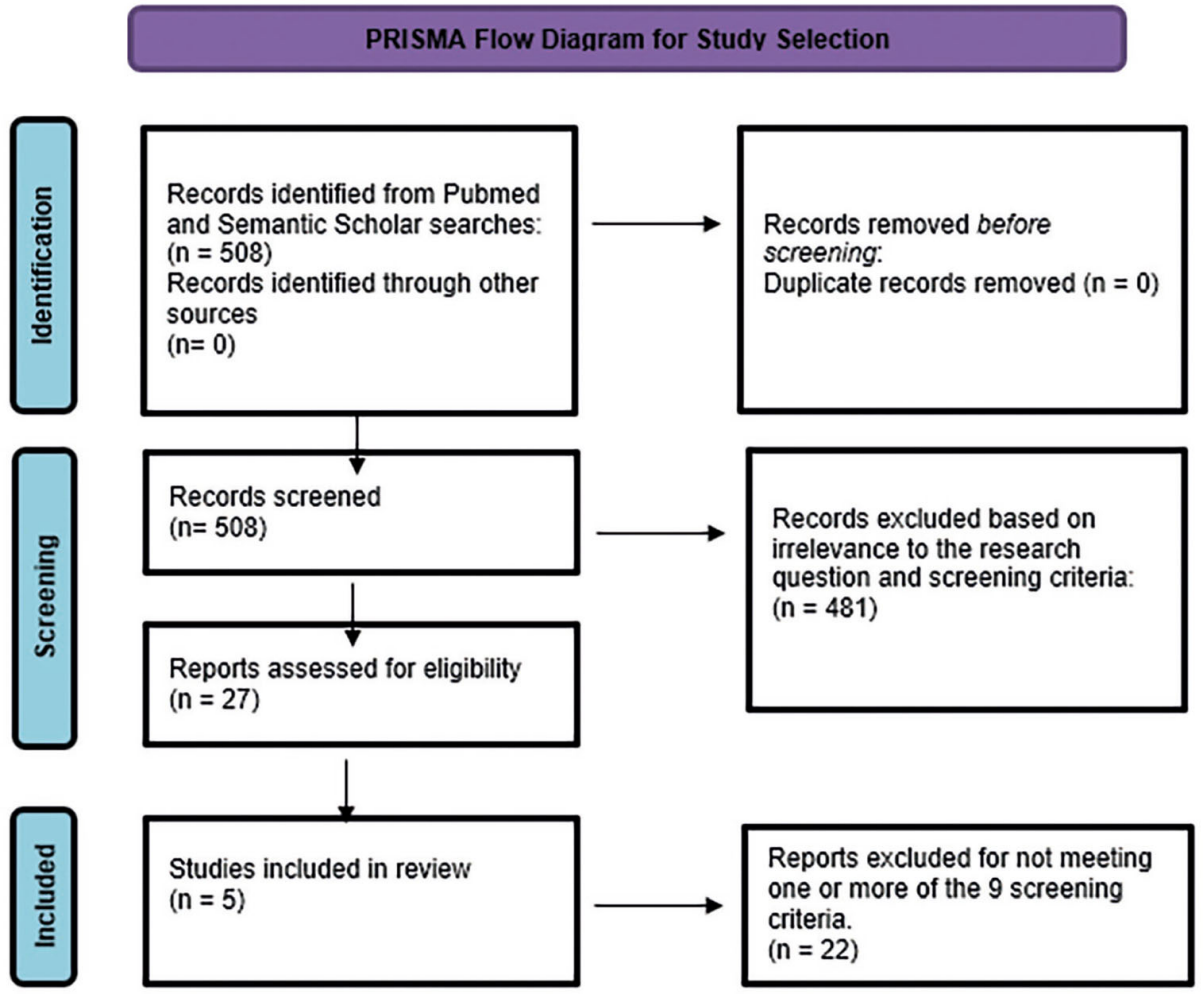
Flowchart according to PRISMA guidelines

This systematic review was conducted to evaluate the role of montelukast (Singulair®) in the prevention and treatment of Baker Grade III-IV capsular contracture in patients with breast implants. The review adhered to the Preferred Reporting Items for Systematic Reviews and Meta-Analyses (PRISMA) guidelines to ensure methodological rigor and transparency (Fig. 1). Institutional review board (IRB) approval was not required, as the study was retrospective and involved the collection of existing and publicly available data from previously published studies. However, all research adhered to the ethical principles outlined in the Declaration of Helsinki and written informed consent was provided by all patients.

### Search Strategy and Information Sources

A comprehensive literature search was performed using the two major databases PubMed and Semantic Scholar to identify relevant studies published in English between January 2000 and February 2025. The search strategy utilized Boolean operators (AND, OR) and Medical Subject Headings (MeSH) terms to refine results, incorporating a combination of the following keywords: Montelukast, Singulair, capsular contracture, Baker grade, and breast implants. Where appropriate, free text terms were truncated to include relevant alternate word endings. Only studies that reported original clinical data, including randomized controlled trials (RCTs), prospective observational studies, retrospective cohort studies, and case reports, were considered eligible for inclusion. Studies that involved animal models, *in vitro* research, or other preclinical work were excluded. Additionally, studies that did not report key clinical outcomes, such as changes in Baker grade or revision surgery rates, were not considered for inclusion in the final analysis.

### Screening and Study Selection

The initial search retrieved a total of 508 articles, which were independently screened by two reviewers to ensure methodological rigor and minimize bias. Any discrepancies in the inclusion process were resolved through consensus discussion. After an initial title and abstract screening, 27 studies underwent a full-text review to assess their eligibility. However, only five studies met all predefined inclusion criteria and were ultimately included in the systematic review and meta-analysis (Fig. 1). To be considered eligible, a study needed to investigate montelukast or its brand name, Singulair®, as a standalone intervention for the prevention or treatment of capsular contracture. Additionally, it was required to report outcomes specifically related to Baker Grade capsular contracture and include a comparison group in which montelukast was compared to either a placebo, no treatment, or standard care. A minimum follow-up period of six months was required to ensure adequate observation of clinical outcomes. The study had to be designed as an RCT, prospective or retrospective cohort study, or case–control study, and montelukast had to be administered independently, without combination with other experimental treatments. The severity of capsular contracture had to be measured using the Baker classification. Moreover, only studies published after December 1999 and written in English language were included in the final analysis. The diagnosis and grading of capsular contracture were based on the Baker classification, which remains the standard clinical system for assessing capsular firmness and deformity. In this scale, Grade I represents a soft, natural breast with no contracture, Grade II indicates a slightly firm breast with normal appearance, Grade III corresponds to a firm breast with visible distortion, and Grade IV denotes a hard, painful, and clearly deformed breast [10, 11]. This classification was applied consistently by the evaluating surgeon at each follow-up visit for both the retrospective case series and the studies included in the systematic review.

The diagnosis of capsular contracture was primarily clinical, based on the Baker classification, and was supported by imaging when indicated. In all patients with suspected or recurrent contracture, diagnostic ultrasound was performed to assess implant integrity and capsular thickness. In selected cases with inconclusive ultrasound findings or complex postoperative anatomy, magnetic resonance imaging (MRI) was used to confirm capsular fibrosis and exclude implant rupture. Mammography was not routinely performed, as MRI and ultrasound provided sufficient diagnostic accuracy for this patient population [12].

Treatment response and clinical improvement were evaluated using both objective and subjective parameters. Clinically, improvement was defined as a reduction of at least one grade on the Baker classification scale, decreased breast firmness, and resolution of pain or discomfort reported by the patient. Imaging confirmation was obtained by ultrasound or MRI, demonstrating reduced capsular thickness and normalization of implant contour when available. Patient satisfaction and absence of capsular recurrence during follow-up were also considered as indicators of successful response.

### Data Extraction and Bias Assessment

Relevant information was subsequently extracted using a pre-piloted data extraction form. The following information was extracted: authors, publishing year, study design, total sample size, sample size with montelukast treatment, intervention protocol, follow-up duration, outcome, main result, and main conclusion.

To evaluate the risk of bias in the included studies a structured assessment using established bias evaluation tools was used. For randomized controlled trials, the Cochrane Risk of Bias Tool was applied. Non-randomized studies, including cohort and case–control studies, were assessed using the Newcastle–Ottawa Scale (NOS) [13, 14]. Disagreements between reviewers were resolved through discussion, and if necessary, consultation with a third reviewer. The final NOS score was converted to Agency for Healthcare Research and Quality (AHRQ) standards to assess whether a study was of good, fair or poor quality. Studies that were determined to have a high risk of bias were not excluded but were considered separately in the interpretation of results.

## Results

A total of five studies met the inclusion criteria and were included in the systematic review (Table 1). The studies varied in design, sample size, follow-up duration, and patient populations, but all provided data relevant to the effect of montelukast on capsular contracture in breast implant patients. For all included studies, Baker Grade classification was the primary outcome used to assess the severity of capsular contracture, providing a consistent parameter for comparison. Study populations and follow-up durations varied considerably among the included studies, with a combined total of 1343 patients across all studies. Sample sizes ranged from 19 to 1122 patients, and follow-up periods ranged from five months to two years. Most studies focused on patients undergoing primary cosmetic breast augmentation or revision surgery for existing capsular contracture, with both smooth and textured implants represented.

**Table 1:** Literature overview

Paper	Study design	Study population	Intervention protocol	Follow-up	Results
Effects of singulair (montelukast) treatment for capsular contracture [7]	Retrospective cohort study	Patients: 19 Gender: not stated, likely female Mean Age: 44.2y Implant type: not stated Previous CC: 2 patients	Montelukast 10mg/d for 3 months (start 1.POD)	5–36 months	19 patients, 21 breasts. Mostly secondary CC. 12/19 improved, 7 fully resolved. Baker I-II responded better than Baker III-IV. All Baker III-IV needed revision. 2 prophylactic cases, no CC. Significant improvement for lower Baker grades (p < 0.05).
Prevention of capsular contracture using leukotriene antagonists [6]	Prospective cohort study	Patients: 82 Gender: not stated, likely female Mean Age: 33y Implant type: textured silicone Previous CC: not stated	Montelukast 10mg/d for 3 months (start 8.POD)	24 months	82 patients, 164 breasts. 7/74 (9.5%) in Montelukast group developed CC (all Baker II). 19/90 (21%) in non-Montelukast group (15 Baker II, 3 Baker III, 1 Baker IV). Montelukast reduced CC risk (p < 0.05). Most CC cases Baker II.
Prophylactic leukotriene inhibitor therapy for the reduction of capsular contracture in primary silicone breast augmentation: experience with over 1100 cases [11]	Retrospective cohort study	Patients: 1122 Gender: all female Age: not stated Implant type: smooth silicone Previous CC: not stated	Montelukast 10mg/d for 3 months (start 1.POD)	12 months	1122 patients, 1-year follow-up. Montelukast reduced CC rates, but not significant. Accolate showed significant CC reduction (p < 0.05). Some Baker III-IV downgraded to Baker II after extended Montelukast (additional 3 months).
The Potential benefit of preemptive leukotriene inhibitor treatment to breast augmentation/mastopexy surgery. [10]	Prospective cohort study	Patients: 72 Gender: not stated Mean Age: 32 +/-4 y Implant type: smooth saline Previous CC: not stated	Montelukast 10mg/d for 3 months (start 8.POD)	7.5–15.5 months	72 patients, follow-up ~11 months. All smooth saline implants. Group A (Montelukast) 0 cases Baker III-IV. Group B (no Montelukast) 4 cases Baker III-IV (11.4%). Trend toward lower CC with Montelukast (p = 0.0509).
Use of leukotriene receptor antagonists for preventive maintenance of breasts capsular contracture [12]	Prospective cohort study	Patients: 48 Gender: all female Age: mean 32y Implant type: silicone cohesive gel, smooth & textured Previous CC: not stated	Montelukast 10mg/d for 6 weeks (start 1.POD)	12 and 24 months	48 patients, 2-year follow-up. After 1 year (montelukast group): 71% Baker I, 29% Baker II. Comparison group: 42% Baker I, 50% Baker II, 8% Baker III. After 2 years: 38% Baker I, 54% Baker II, 8% Baker III. Higher Baker grades linked to thicker capsules and inflammation.

The prophylactic use of montelukast was evaluated in three of the five studies [8, 15, 16], all of which reported a lower incidence of capsular contracture in patients receiving montelukast compared to untreated patients. In the study by Graf et al., 9.5% of breasts in the montelukast group developed capsular contracture, compared to 21% in the untreated group with a minimum follow-up of 2 years [8]. All capsular contracture cases in the montelukast group were classified as Baker Grade II, with no cases of more severe Baker Grade III or IV contracture [8]. Similarly, Lille and Jacoby reported no cases of Baker Grade III or IV capsular contracture in the montelukast group, while 11.4% of untreated patients developed higher-grade capsular contracture with a mean postoperative follow-up of 11 ± 4.5 months [15]. In contrast, the study by Bresnick observed a non-significant trend toward lower capsular contracture rates in women undergoing primary silicone gel breast augmentation and receiving montelukast compared to untreated patients [16]. However, patients receiving a different leukotriene receptor antagonist, zafirlukast (Accolate®), experienced a statistically significant reduction in capsular contracture rates (*p* < 0.05). The study by Huang and Handel specifically investigated the use of montelukast for the treatment of existing capsular contracture, with follow-up ranging from 5 to 36 months [9]. Improvement in capsular contracture was reported in 63% of patients, with complete resolution observed in 3%. Patients with Baker Grade I or II contracture responded better to montelukast than those with Baker Grade III or IV contracture. All patients with Baker Grade III or IV contracture ultimately required revision surgery, even if they experienced some initial improvement with montelukast. Overall, montelukast was associated with statistically significant improvement in capsular contracture (*p* < 0.05), but its effect was largely limited to lower Baker Grades [16]. The available evidence suggests that montelukast may reduce the incidence of capsular contracture, particularly for Baker Grade I and II cases, when used prophylactically in the early postoperative period. For patients with established capsular contracture, montelukast may provide symptomatic improvement and limited downgrading of Baker Grade, but it appears to be insufficient as a standalone treatment for Baker Grade III or IV capsular contracture, where surgical revision remains necessary. The strongest evidence for the efficacy of montelukast was found in its prophylactic use, particularly when combined with other perioperative management strategies.

The included studies also provided data on the safety and tolerability of montelukast in the context of breast implant surgery. Across all five studies, montelukast was generally well tolerated, with only isolated cases of mild adverse events reported. Graf et al. documented two patients experiencing adverse effects, one with skin lesions and one with epigastric pain, both of which resolved upon discontinuation or with supportive treatment [8]. Bresnick et al. noted that 2.5% of patients discontinued montelukast, primarily due to minor side effects or cost concerns, but no serious drug-related complications were reported [16]. No significant adverse events were observed in several other studies [9, 15]. Overall, the data suggest that montelukast is a well-tolerated medication with a favorable safety profile, supporting its potential role as a safe option for both prevention and adjunctive treatment of capsular contracture in breast implant patients.

### Case Series

In addition to the systematic review, a retrospective case series was conducted to assess the efficacy of adjuvant montelukast in preventing and treating Baker grade III-IV capsular contracture in patients undergoing breast implant surgery. All patients underwent implant exchange with partial capsulectomy and/or capsulotomy and were informed about the potential risks associated with the off-label use of montelukast. Postoperatively, patients were prescribed 10 mg of montelukast orally daily for 90 days and instructed to perform breast massage twice daily. Side effects, histological findings, and radiological assessments were recorded. Breast firmness was evaluated at each follow-up visit by the senior surgeon using the Baker classification.

#### Case 1

**Fig. 2:**
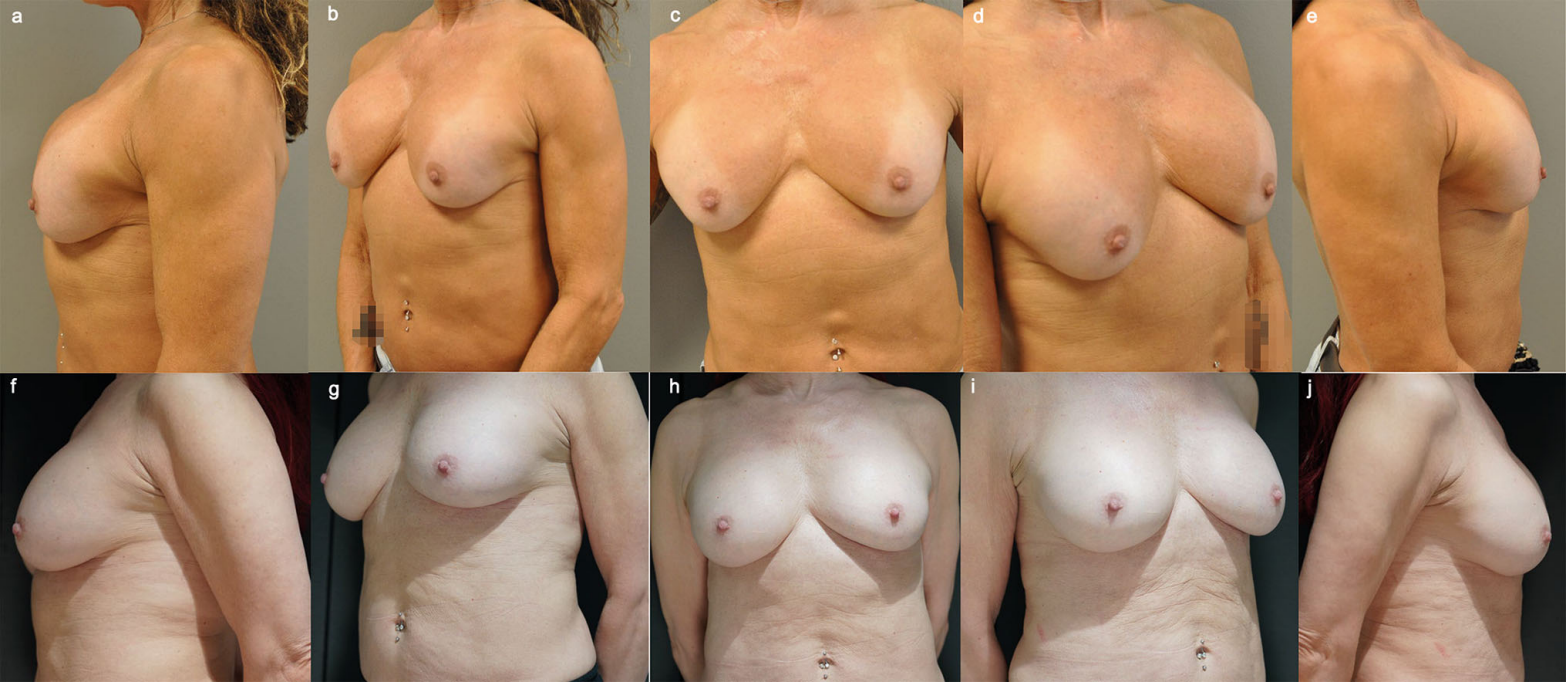
A 61-year-old female patient presented with a grade IV Baker’s capsule after a breast augmentation more than 20 years ago (**a**–**e**). Final follow-up after 22 months (**f**–**j**)

A 61-year-old female patient presented with Baker grade IV capsular contracture following submuscular breast augmentation performed over 20 years before with 300 cc microtextured silicone implants (SILTEX® Mentor®). In February 2023, she underwent partial capsulectomy and implant exchange with smooth, round, medium-profile silicone implants (350 cc, submuscular, Eurosilicone®). Capsular fibrosis was confirmed both by preoperative magnetic resonance imaging (MRI) and histologically. Postoperatively, montelukast 10 mg daily was prescribed for three months. During follow-up, a residual symptomatic siliconoma was identified and subsequently removed in October 2024, along with an implant exchange. At the time of revision surgery, the breast capsule appeared thin and unremarkable, with no macroscopic signs of capsular contracture, consistent with MRI findings and histological analysis (Fig. 2).

#### Case 2

**Fig. 3:**
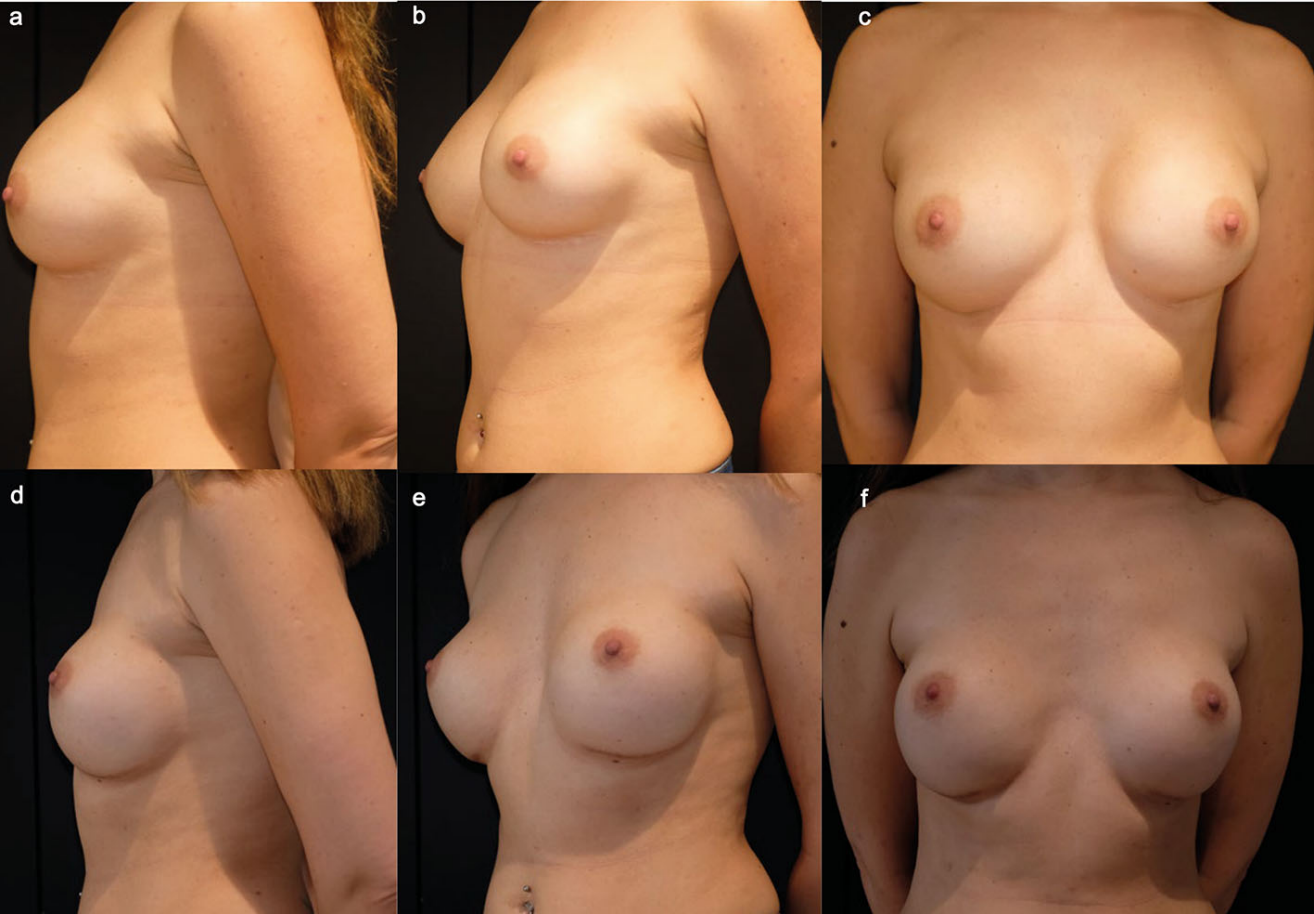
A 42-year-old female patient presented with a grade III Baker's capsule fibrosis after breast augmentation in 2007 (**a**–**c**). Final follow-up after 36 months (**d**–**f**)

A 42-year-old female patient presented with Baker grade III capsular contracture following submuscular breast augmentation performed in 2007 with 290 cc textured silicone implants (Meme® TMP, Polytech®). In May 2022, she underwent partial capsulectomy, capsulotomy, and implant exchange with smooth, high-projection silicone implants (315 cc, submuscular, polySMOOTH™, Polytech®). Capsular fibrosis was confirmed both by MRI and histologically. Postoperatively, montelukast 10 mg daily was prescribed for three months, which the patient tolerated well. During the follow-up period, a wound healing complication developed in the right submammary region, requiring revision under local anesthesia. At the 36-month follow-up, the patient remained asymptomatic, with no clinical or radiological evidence of recurrent capsular contracture (Fig. 3).

#### Case 3

**Fig. 4:**
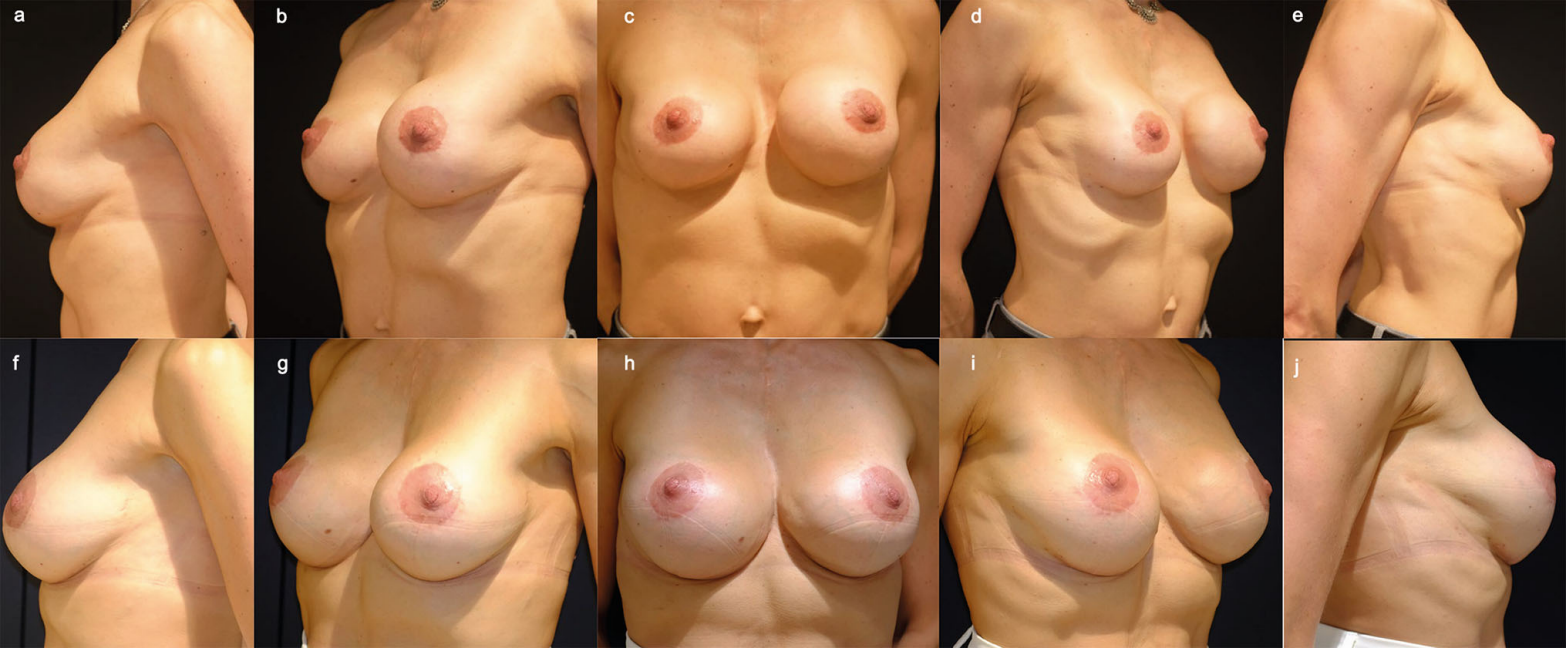
A 47-year-old female patient presented with a grade III Baker’s capsule fibrosis after an epimuscular breast augmentation in 2012 (**a**–**g**). Final follow-up after 24 months (**h**–**j**)

A 47-year-old female patient presented with Baker grade III capsular contracture following subglandular breast augmentation in 2012 with 255 cc (McGhan®). In November 2022, she underwent partial capsulectomy, capsulotomy, and implant and pocket exchange to submuscular (Meme® XP 285cc right side, Meme HP® 275cc left side). Capsular contracture was confirmed both by MRI and histologically. Postoperatively, montelukast 10 mg daily was prescribed for three months, which the patient tolerated without any adverse effects. At the 24-month follow-up, she remained asymptomatic with no clinical or radiological (MRI) evidence of recurrent capsular contracture (Fig. 4).

## Discussion

The incidence of capsular contracture varies across studies, but it consistently ranks as one of the most common reasons for breast implant revision surgery. The exact molecular mechanism behind capsular contracture is not completely understood, although it is generally agreed that an inflammatory response causes periprosthetic scar formation. Leukotriene receptor antagonists are known to alter the inflammatory cascade and likely aid in the prevention of fibrosis associated with capsular contracture [1, 2]. Risk factors identified include the use of smooth implants, subglandular placement, silicone-filled implants, previous radiotherapy to the breast, postoperative bleeding, and infection [4, 17].

The primary treatment for established capsular contracture is surgical intervention, typically involving capsulectomy. The choice between total and partial capsulectomy depends on the thickness of the capsule. For thicker capsules, total capsulectomy is generally recommended, while partial capsulectomy may be sufficient for thinner capsules [18].

In patients with very thin, soft tissue coverage, particularly after mastectomy, capsulectomy may be risky, and full-thickness capsulotomies are often sufficient [19]. This is especially true in submuscular implant placement, where posterior capsulectomy carries a risk of pleural injury and pneumothorax due to the close proximity to the chest wall and pleura [20].

Leukotrienes—namely, LTC4, LTD4, and LTE4—are implicated in the inflammatory cascade and have been postulated to play a role in the development of capsular contracture [3]. Histologic analysis of contracted (Baker score II–IV) capsules revealed up-regulation of one of the leukotriene receptors (cysLT1), as compared to controls [2].

Montelukast, originally approved for asthma and allergic rhinitis management, has gained attention for its possible off-label use in addressing capsular contracture [21]. Singulair® selectively inhibits LTD4, which is the most active of the leukotrienes. By blocking leukotriene receptors, it may reduce inflammation and fibrosis. Therefore, leukotriene antagonists Accolate® and Singulair® have been prescribed by plastic surgeons off-label to treat and prevent capsular contracture [3].

Most research on the efficacy of leukotriene antagonists has been with Accolate®. Two recent studies in a rat model showed that administration of Accolate® in the presence of textured silicone implants resulted in a statistically significant decrease in mean capsule thickness when compared to controls [8, 9]. There was, however, no difference in capsule thickness with smooth implants.[8] In a prospective study that measured mammary compliance, Scuderi et al. found an increase in breast compliance of up to 24% after six months in patients treated with Accolate® compared to controls [22, 23]. In another study, Reid et al. found that 55% of patients with capsular contracture had complete resolution of symptoms and 24% had partial reduction of symptoms after taking Accolate® for six months [24]. However, the use of Accolate® has largely been abandoned because of potential adverse effects on the liver, including hepatitis and liver failure [4].

In another study by Huang et al., the authors reviewed a series of patients treated with Singulair® to determine whether it improved capsular contracture after breast implant surgery [9]. Their preliminary results showed that Singulair® improved capsular contracture. Breasts with mild capsular contracture (Baker score < III) appeared to have better improvement with Singulair® compared to those with more severe contracture (Baker score III and IV). Thus, the authors concluded that Singulair® is well tolerated with minimal side effects and can be administered to patients after breast implant surgery to improve contracture [9]. Most common reported side effects are headache, influenza-like symptoms, abdominal pain, cough, and dyspepsia [25].

Our preliminary case series goes in line with those previous studies: At final follow-up after surgery, none of the patients developed capsular contracture and both the surgeon and patient were pleased with the result. Importantly, there were no reported side effects. That being said, it remains to be determined whether Singulair® alone and/or breast massage reduces the risk of capsular contracture. Nevertheless, Singulair® has a potential benefit in capsular contracture after partial or total capsulectomy and we had no signs of recurrence after the surgery. Singulair® is well tolerated with minimal side effects and can be administered to patients after breast implant surgery to improve capsular contracture.

Other described conservative treatments for capsular contracture include implant massage, ultrasound therapy, and noninvasive shock wave treatment, although reporting varying degrees of efficacy [25, 26].

Ultimately, our study is limited by a low number of patients, a lack of homogeneity and with limited follow-up compared to other studies. Therefore, evaluation of a larger cohort in a prospective randomized double-blind controlled trial with negative controls is required together with patient-reported outcome measurements (PROM) such as BREAST-Q questionnaire to confirm our findings [27]. Lastly, statistical analysis should be employed to determine whether Singulair® significantly reduces the risk of developing capsular contracture and whether there are specific patient factors (such as smoking or diabetes) associated with treatment response.

## Conclusion

This systematic review and case series provide further evidence supporting the potential role of montelukast in preventing and managing capsular contracture. The data suggest that montelukast is most effective in prophylaxis and in mild to moderate contracture cases, while severe cases (Baker grade III-IV) still require surgical intervention. Additionally, our findings confirm that montelukast is well tolerated, with no significant adverse effects. The integration of this therapy into clinical practice could potentially reduce the incidence of recurrent capsular contracture, improve patient outcomes and decrease the need for revision surgeries. Further research is needed to establish standardized treatment protocols, determine the optimal duration and dosage of montelukast, and evaluate its long-term impact on capsular formation and fibrosis.
